# Nuclear lamina invaginations are not a pathological feature of C9orf72 ALS/FTD

**DOI:** 10.1186/s40478-021-01150-5

**Published:** 2021-03-19

**Authors:** Alyssa N. Coyne, Jeffrey D. Rothstein

**Affiliations:** 1 Brain Science Institute, Johns Hopkins University School of Medicine, Baltimore, MD 21205 USA; 2 Department of Neurology, Johns Hopkins University School of Medicine, Baltimore, MD 21205 USA

**Keywords:** C9orf72, Nuclear morphology, Nucleocytoplasmic transport, Nuclear pore complex, Lamin B1, Nuclear envelope, Amyotrophic Lateral Sclerosis, Frontotemporal Dementia

## Abstract

The most common genetic cause of familial and sporadic amyotrophic lateral sclerosis (ALS) is a GGGGCC hexanucleotide repeat expansion (HRE) in the C9orf72 gene. While direct molecular hallmarks of the C9orf72 HRE (repeat RNA foci, dipeptide repeat protein pathology) are well characterized, the mechanisms by which the C9orf72 HRE causes ALS and the related neurodegenerative disease frontotemporal dementia (FTD) remain poorly understood. Recently, alterations to the nuclear pore complex and nucleocytoplasmic transport have been accepted as a prominent pathomechanism underlying C9orf72 ALS/FTD. However, global disruptions to nuclear morphology and the nuclear lamina itself remain controversial. Here, we use a large number of induced pluripotent stem cell derived spinal neurons and postmortem human motor cortex sections to thoroughly examine nuclear morphology and nuclear lamina disruptions with light microscopy. In contrast to previous studies in artificial overexpression model systems, endogenous levels of the C9orf72 HRE do not increase the frequency of nuclear lamina invaginations. In addition, the C9orf72 HRE has no impact on overall nuclear shape and size. Notably, the frequency of nuclear Lamin B1 invaginations increases with cellular aging, independent of the C9orf72 HRE. Together, our data suggest that nuclear morphology is unaltered in C9orf72 ALS/FTD.

**Supplementary Information:**

The online version contains supplementary material available at 10.1186/s40478-021-01150-5.

## Introduction

Amyotrophic lateral sclerosis (ALS) and Frontotemporal Dementia (FTD) comprise a spectrum of neurodegenerative diseases sharing both pathological and genetic underpinnings. A hexanucleotide repeat expansion (HRE) in the C9orf72 gene has recently been identified as the most common genetic cause of ALS [5, 28]. This intronic GGGGCC (G_4_C_2_) repeat has been shown to generate toxic G_4_C_2_ and G_2_C_4_ RNA species which on occasion can accumulate into nuclear and rare cytoplasmic RNA foci. Further, these repeat RNAs can be translated via non-canonical repeat associated non-ATG (RAN) translation to generate five distinct dipeptide repeat proteins (DPRs; poly(GA), poly(GR), poly(GP), poly(PA), poly(PR)) which accumulate into nuclear and cytoplasmic aggregates in postmortem patient tissue and multiple cellular and animal model systems [8]. While these molecular hallmarks of the C9orf72 HRE have been well described, the mechanisms by which they elicit disease remain poorly understood.

Recently, disruptions to the nuclear pore complex (NPC) and nucleocytoplasmic transport (NCT) have been uncovered as a prominent and early pathomechanism underlying C9orf72 ALS/FTD [3, 7, 14, 36]. While G_4_C_2_ repeat RNA appears to coordinate alterations to NPC composition ultimately culminating in defective NCT [3], DPRs have independently been shown to impact NCT via interactions with nuclear transport receptors [10, 11, 19, 32]. Intriguingly, a recent study suggests that a reduction in specific nucleoporins (Nups) can disrupt the nuclear lamina network in mouse embryonic fibroblasts. Conversely, knockdown of lamins impacted overall NPC number [16]. Given the role of the nuclear lamina in the compartmentalization of the nucleus and cytoplasm as well as the organization and coordination of nuclear functions [9], it has been hypothesized that the C9orf72 HRE can also impact global nuclear morphology. Multiple reports have suggested that nuclear lamina disruptions are a prevalent pathological feature of C9orf72 ALS/FTD [7, 18, 24, 37]. However, many of these studies were conducted in non-neuronal cells [7, 18] or in the context of highly artificial overexpression of G_4_C_2_ repeats or individual DPRs [7, 18, 37]. In a recent study employing a small number of induced pluripotent stem cell (iPSC) lines, the authors showed that nuclear invaginations were not more frequent in C9orf72 iPSNs compared to controls [24]. Thus, there still exists much controversy as to whether nuclear lamina disruptions are a real C9orf72 HRE pathology, despite its continued used as a phenotypic readout in many studies.

Using iPSC derived spinal neurons (iPSNs) and postmortem human motor cortex, we used immunostaining and light microscopy to thoroughly examine nuclear morphology and the nuclear lamina in human neurons expressing endogenous levels of the C9orf72 HRE. In contrast to previous overexpression based studies, we do not observe an increase in the frequency of nuclear lamina invaginations. In fact, the number of Lamin B1 invaginations increased with cellular age, independent of the presence of the C9orf72 HRE. Further, overall nuclear morphology and volume are unaffected in C9orf72 neurons. Together, our data reveal that alterations in nuclear morphology and the nuclear lamina are not a pathological consequence of the C9orf72 HRE.

## Materials and methods

### iPSC derived neuronal differentiation

*C9orf72* and non-neurological control iPSC lines were obtained from the Answer ALS repository at Cedars-Sinai (see Additional file 5: Supplemental Table 1 for demographics). iPSCs were maintained on Matrigel with MTeSR, maintained according to Cedars Sinai SOP, and differentiated into spinal neurons using the direct induced motor neuron (diMNs) protocol as previously described [3]. All cells were maintained at 37 °C with 5% CO_2_. iPSCs and iPSNs routinely tested negative for mycoplasma.

### Nuclei isolation and super resolution structured illumination microscopy

Nuclei were isolated from iPSNs and postmortem human motor cortex tissue using the Nuclei Pure Prep Nuclei Isolation Kit (Sigma Aldrich) following manufacturer protocol with slight modifications as previously described [3]. About 100–150 mg of frozen postmortem motor cortex tissue (obtained from the Target ALS Human Postmortem Tissue Core (see Additional file 5: Supplemental Table 2 for demographic information) was used for nuclei isolation. A 1.85 M sucrose gradient was used to enrich for neuronal nuclei. Following isolation, nuclei were centrifuged onto collagen coated (1 mg/mL; Advanced Biomatrix) slides with a CytoSpin 4 centrifuge (Thermo Fisher Scientific) and immunostained as previously described [3]. Antibodies for immunostaining are as follows: 1:500 Rabbit Anti-Lamin B1 (Abcam ab16048), 1:500 Chicken Anti-NeuN (Millipore ABN91), 1:1000 Goat Anti-Rabbit Alexa 488 (Invitrogen A11034), 1:1000 Goat Anti-Chicken Alexa 647 (Invitrogen 21,449). Isolated iPSN nuclei were subsequently imaged by super resolution structured illumination microscopy (SIM) using a Zeiss ELYRA S1 as previously described [3]. All images were acquired using identical imaging parameters (e.g. laser power, gain). Representative images are presented as 3D maximum intensity projections generated in Zeiss Zen Black 2.3 SP1. Isolated nuclei from postmortem motor cortex tissue were imaged by confocal microscopy using a Zeiss LSM 980. All images were acquired using identical imaging parameters. Representative images are presented as maximum intensity projections generated in Zeiss Zen Blue 2.3 lite. Images were faux colored green for contrast and display.

### Immunostaining and confocal imaging of iPSNs

On day 12 of differentiation, iPSNs were plated in 24 well optical bottom plates (Cellvis). At day 32 of differentiation, iPSNs were fixed and immunostained as previously described [3]. Antibodies for immunostaining are as follows: 1:500 Rabbit Anti-Lamin B1 (Abcam ab16048), 1:1000 Guinea Pig Anti-Map2 (Synaptic Systems 188,004), 1:1000 Goat Anti-Rabbit Alexa 488 (Invitrogen A11034), 1:1000 Goat Anti-Guinea Pig Alexa 647 (Invitrogen A21450). iPSNs were imaged using a Zeiss LSM 800 confocal microscope. All images were acquired using identical imaging parameters (e.g. laser power, gain). Unless otherwise indicated, images presented are maximum intensity projections generated in Zeiss Zen Blue 2.3.

### Human tissue immunofluorescence

Non-neurological control and *C9orf72* patient postmortem paraffin embedded motor cortex sections were obtained from the Target ALS Human Postmortem Tissue Core (see Additional file 5: Supplemental Table 2 for demographic information). Antigen retrieval and immunofluorescent staining was conducted as previously described [3]. Antibodies for immunostaining are as follows: 1:500 Rabbit Anti-Lamin B1 (Abcam ab16048), 1:1000 Guinea Pig Anti-Map2 (Synaptic Systems 188,004), 1:1000 Goat Anti-Guinea Pig Alexa 488 (Invitrogen A11073), 1:1000 Goat Anti-Rabbit Alexa 647 (Invitrogen A21245). Nuclei from Map2 positive Layer V neurons were imaged with a 63X objective and a Zeiss Axioimager Z2 fluorescent microscope equipped with an apotome2 module. All images were acquired using identical exposure times. Images are presented as default apotome processed images generated in Zeiss Zen Blue 2.3.

### Analysis of nuclear morphology and nuclear lamina invaginations

Nuclear circularity, sphericity, and volume measurements were calculated based on DAPI (confocal imaging) or NeuN (SIM imaging) staining using the Nucleus J plugin in FIJI. With the exception of circularity measurements in postmortem tissue, full z stacks of nuclei were used for analyses. For analysis of Lamin B1 invaginations, images were blinded, and analysis was manually conducted using single z planes (postmortem human tissue) or full z stacks (iPSNs). Only those Lamin B1 alterations that were not visualized as part of the normal curvature/folding of the surface of nuclei were counted as a true invagination.

### Statistical analysis

All data analysis was conducted with FIJI as described above. The analyzer was completely blinded to genotype and passage information. All statistical analyses were performed using GraphPad Prism version 9 (GraphPad). Statistical analyses were performed whereby the average of all nuclei or cells evaluated per each iPSC line or patient represents n = 1. The total number of nuclei or cells evaluated per experiment is indicated in the figure legends. Two-way ANOVA with Tukey’s multiple comparison test or Chi-square test was used as described in figure legends. *p < 0.05, **p < 0.005, *** p < 0.0005, **** p < 0.0001. Violin plots are used to display the full spread and variability of large data sets (> 10 data points). Center dotted line indicates median value. Two additional dotted lines indicate the 25th and 75th percentiles. Stacked bar graphs are used to display summary data for nuclear lamina invagination analyses. Bar graphs with individual data point representing the average of 100 cells or 50 nuclei per iPSC line or patient are used to display summary data in Additional file 1, 2, 3, 4: supplemental figures.

## Results

### Nuclear morphology is impacted by cellular age independent of the C9orf72 HRE in iPSNs

**Fig. 1:**
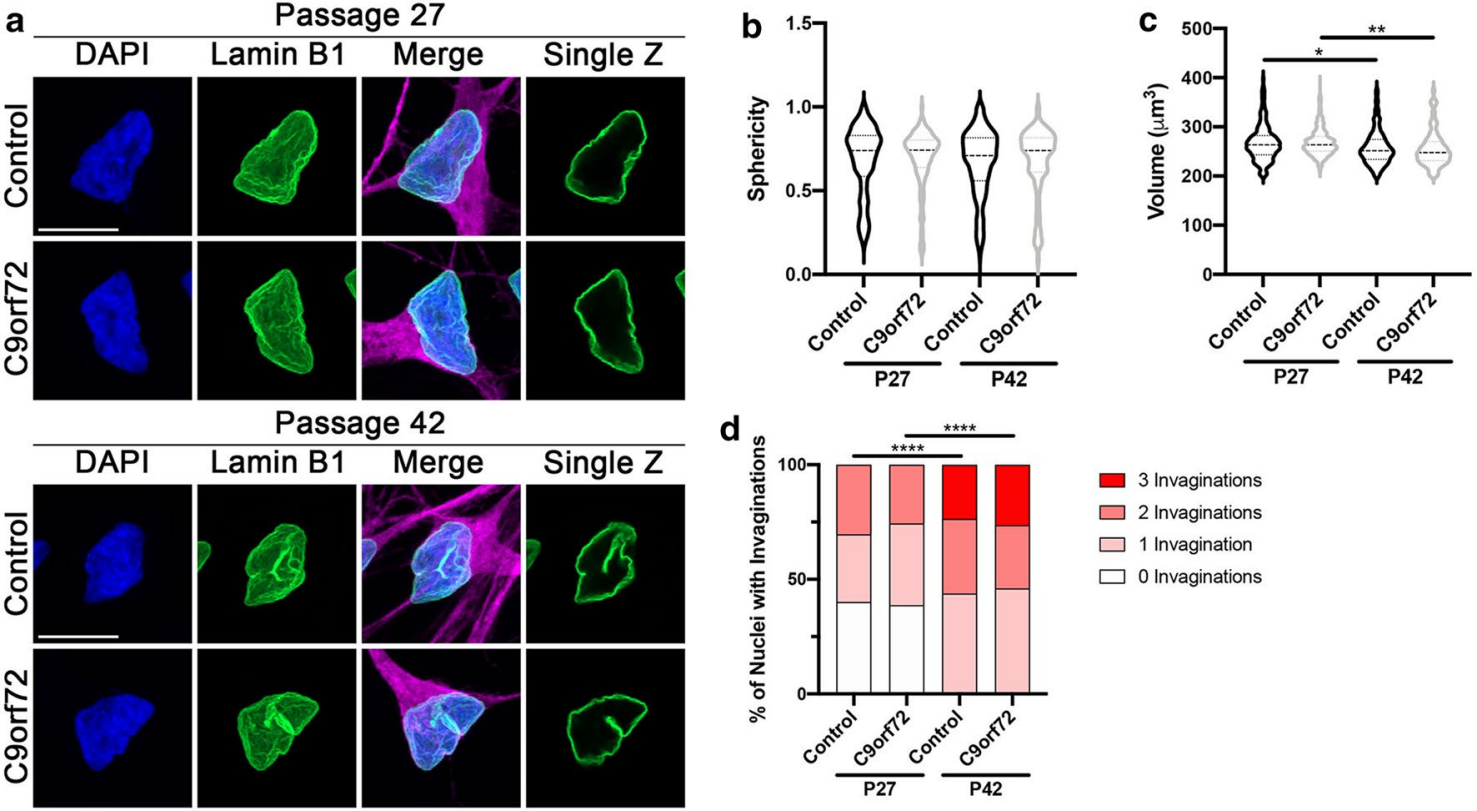
Cellular age increases the frequency of nuclear lamina disruptions independent of the presence of the C9orf72 HRE. **a** Immunostaining and confocal imaging for Lamin B1 in iPSNs at day 32 of differentiation. Genotype as indicated on left, antibody and iPSC passage number as indicated on top. **b, c** Quantification of nuclear sphericity (**b**) and nuclear volume (**c**). n = 8 control and 8 *C9orf72* iPSC lines, at least 100 Map2 + neurons per line and iPSC passage. Two-way ANOVA Tukey’s multiple comparison test was used to calculate statistical significance. *p < 0.05, **p < 0.01. **d** Quantification of number of Lamin B1 invaginations per nucleus. n = 8 control and 8 *C9orf72* iPSC lines, at least 100 Map2 + neurons per line and iPSC passage. Chi-square test was used to calculate statistical significance. ****p < 0.0001. Scale bar = 10 μm

We have previously demonstrated that pathological alterations, including those involving the NPC, identified in iPSNs are also observed in motor cortex autopsy tissue from a large cohort of C9orf72 patients [3, 6]. As opposed to end stage postmortem tissue, iPSCs and iPSNs provide an unparalleled opportunity to examine cellular phenotypes over time as pathological cascades emerge and mature in vitro. Thus, iPSNs are an invaluable tool for studying pathomechanisms of disease in an endogenous genetic setting. To examine nuclear morphology in control and C9orf72 iPSNs from a large number of patients, we employed confocal microscopy of DAPI and Lamin B1 immunostaining based analytics (see Materials and Methods for details). Overall nuclear morphology as measured by sphericity and nuclear volume was comparable between control and C9orf72 iPSN nuclei differentiated from passage matched iPSCs (Fig. 1a–c, Additional file 1: Figure S1A-D). Consistent with the lack of overt changes to global nuclear morphology, we did not observe an increase in the frequency of Lamin B1 invaginations in passage matched control and C9orf72 iPSNs (Fig. 1a, d, Additional file 1: Figure S1E-F). Intriguingly, increased cellular age, which can be initiated by increased iPSC passage number [25], significantly decreased overall nuclear volume and resulted in an increase in the number of nuclear invaginations in both control and C9orf72 iPSNs (Fig. 1a, c, d, Additional file 1: Figure S1E-F). Together, these data suggest that cellular age impacts the frequency of Lamin B1 invaginations independent of the presence of the C9orf72 HRE.

**Fig. 2:**
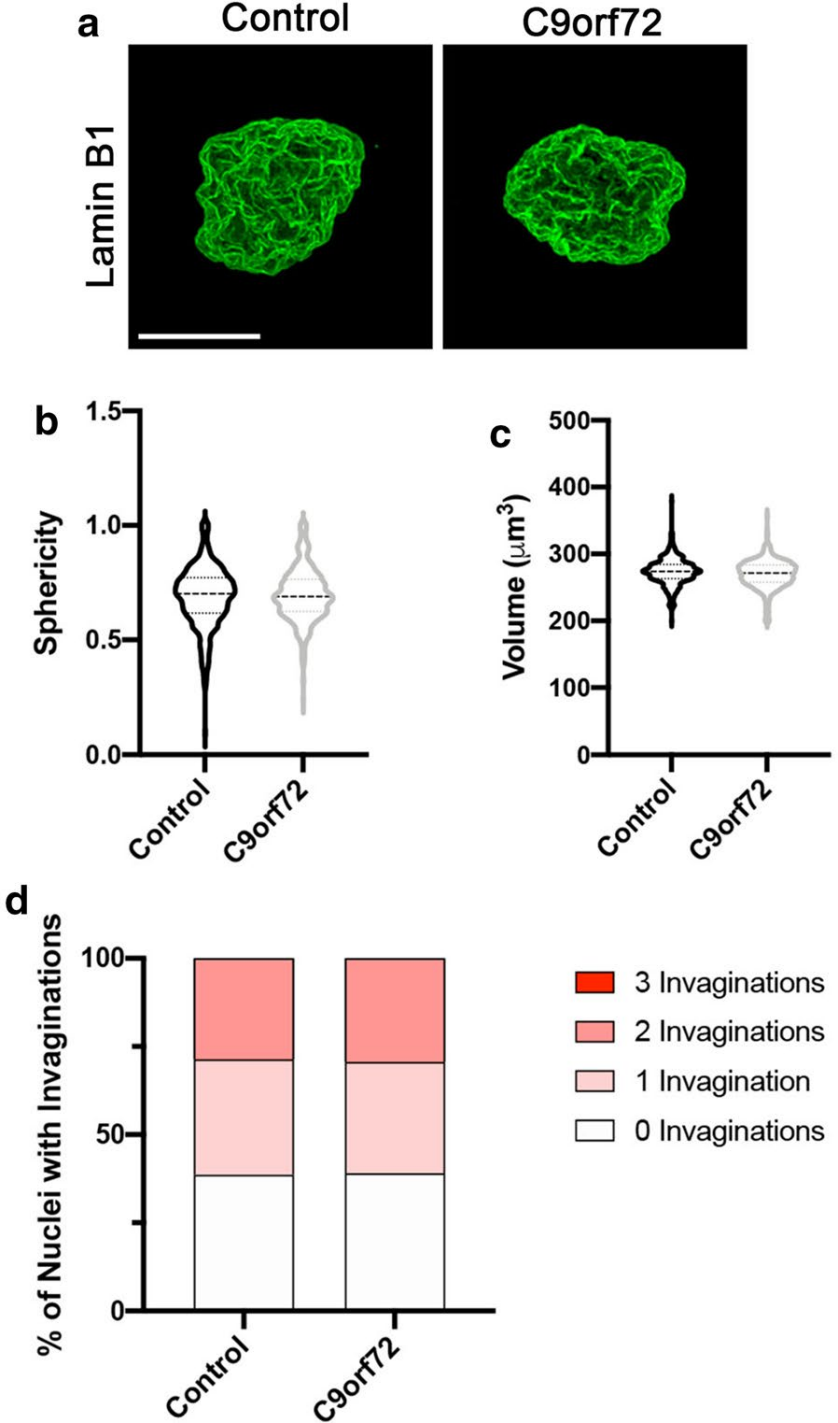
Isolation of iPSN nuclei does not impact nuclear morphology. **a** Maximum intensity projections from SIM imaging of Lamin B1 in nuclei isolated from control and *C9orf72* iPSNs at day 32 of differentiation from passage 27 iPSCs. Genotype as indicated on top. **b, c** Quantification of nuclear sphericity (**b**) and nuclear volume (**c**). n = 10 control and 10 *C9orf72* iPSC lines, at least 50 NeuN + nuclei per line. Student’s t-test was used to calculate statistical significance. **d** Quantification of number of Lamin B1 invaginations per nucleus. n = 10 control and 10 *C9orf72* iPSC lines, at least 50 NeuN + nuclei per line. Chi-square test was used to calculate statistical significance. Scale bar = 5 μm

Nuclear isolation and super resolution microscopy are frequently used to evaluate specific NPC and nuclear pathologies at high resolution [2, 3, 24]. This methodology provides an unparalleled opportunity to visualize nuclear and nuclear envelope proteins using specific antibodies at a resolution of ~ 100 nm [20, 31, 33]. To determine if the process of isolating nuclei disrupts nuclear morphology, we performed immunostaining and super resolution structured illumination microscopy (SIM) for Lamin B1 in nuclei isolated from control and C9orf72 iPSNs. Consistent with our results from confocal imaging of intact iPSNs, we did not observe an effect of the C9orf72 HRE on multiple different measurements of nuclear morphology including sphericity, nuclear volume, or the frequency of Lamin B1 invaginations compared to controls (Fig. 2, Additional file 2: Figure S2). Notably, measurements of all parameters evaluated (sphericity, nuclear volume, number of Lamin B1 invaginations) were similar between whole iPSN confocal and isolated nuclei SIM analytics (Figs. 1b, c, 2b, c, Additional file 1: Figure S1, Additional file 2: Figure S2) suggesting that nuclear isolation does not disrupt nuclear morphology or nuclear envelope integrity.

### Nuclear lamina disruptions are not a pathological hallmark in C9orf72 patient motor cortex

**Fig. 3:**
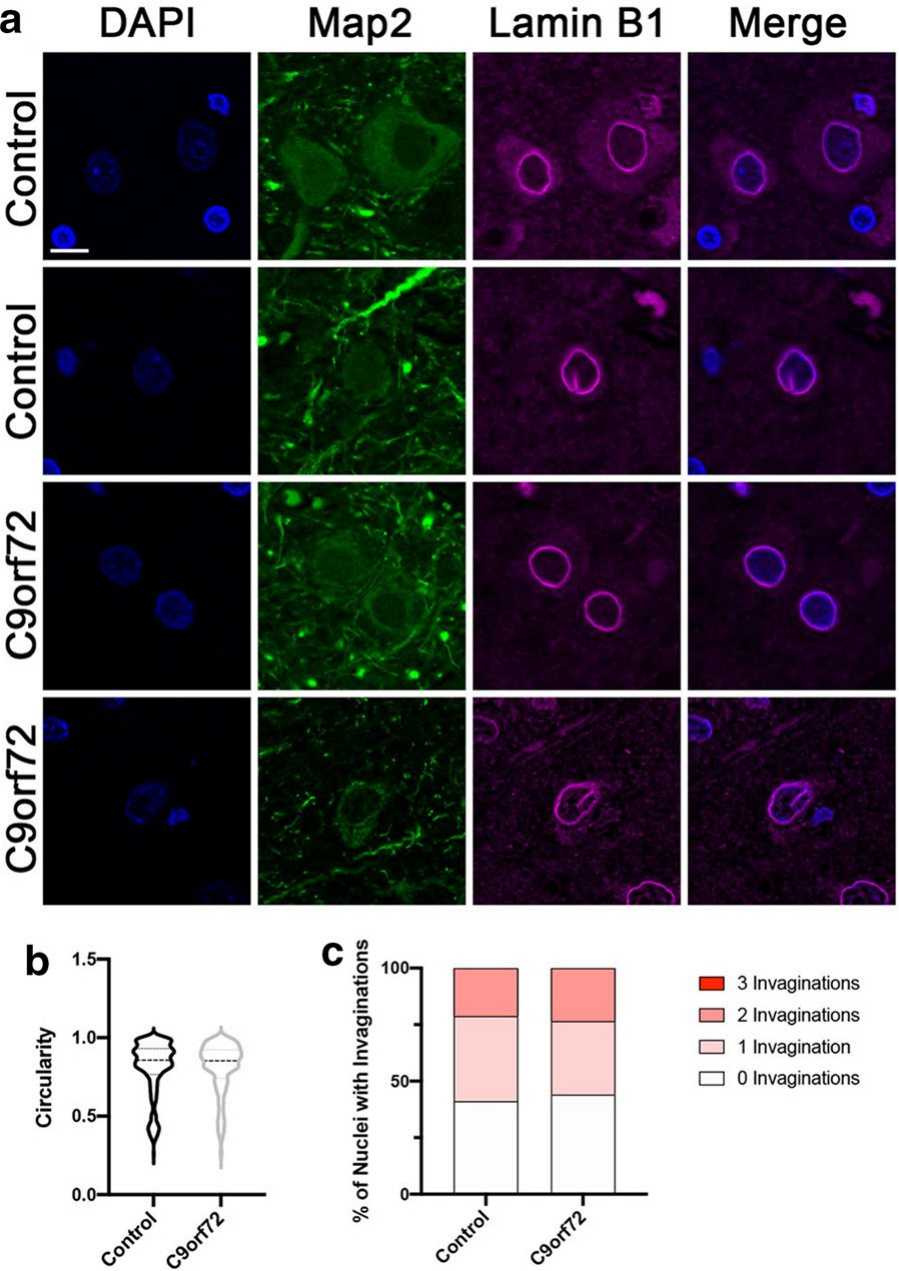
Nuclear lamina invaginations are not a pathological feature of C9orf72 ALS/FTD neurons in postmortem patient motor cortex. **a** Immunostaining for Lamin B1 in postmortem paraffin embedded motor cortex sections. Genotype as indicated on left, antibody as indicated on top. **b** Quantification of nuclear circularity. n = 8 control and 8 *C9orf72* cases, at least 100 Map2 + neurons per case. Student’s t-test was used to calculate statistical significance. **c** Quantification of number of Lamin B1 invaginations per nucleus. n = 8 control and 8 *C9orf72* cases, at least 100 Map2 + neurons per case. Chi-square test was used to calculate statistical significance. Scale bar = 10 μm

**Fig. 4:**
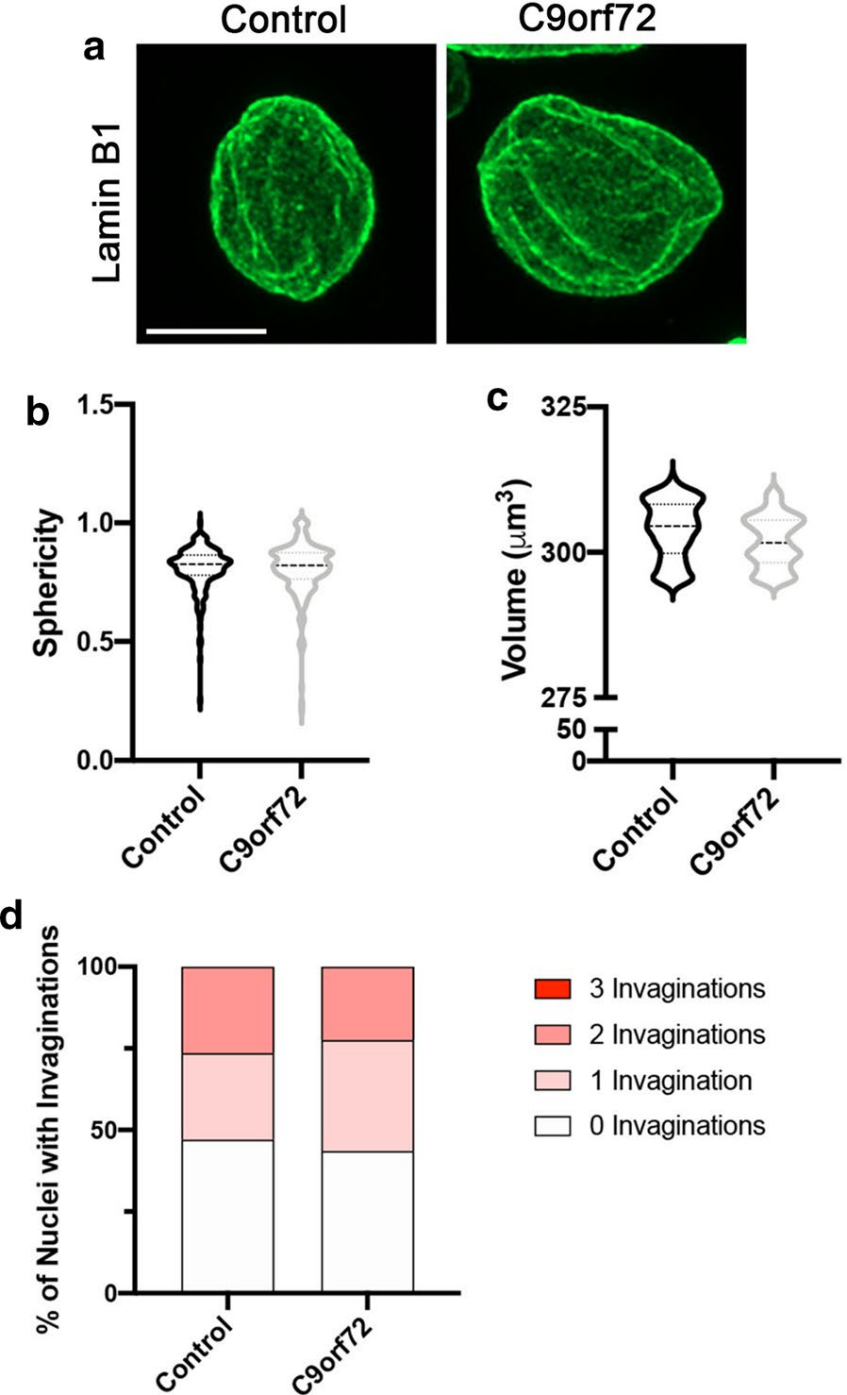
Nuclear lamina invaginations are not a pathological feature in nuclei isolated from C9orf72 ALS/FTD motor cortex. **a** Maximum intensity projections from confocal imaging of Lamin B1 in nuclei isolated from control and *C9orf72* postmortem motor cortex tissue. Genotype as indicated on top. **b, c** Quantification of nuclear sphericity (**b**) and nuclear volume (**c**). n = 4 control and 4 *C9orf72* cases, at least 50 NeuN + nuclei per case. Student’s t-test was used to calculate statistical significance. **d** Quantification of number of Lamin B1 invaginations per nucleus. n = 4 control and 4 *C9orf72* iPSC lines, at least 50 NeuN + nuclei per line. Chi-square test was used to calculate statistical significance. Scale bar = 5 μm

Given our data (Fig. 1) and prior studies suggesting that nuclear lamina invaginations can increase with cellular age [17, 21, 24, 25, 27, 30], we next asked whether Lamin B1 invagination pathology was prevalent in postmortem human motor cortex tissue. Compared to non-neurological controls, we did not observe a difference in nuclear circularity or the frequency of Lamin B1 invaginations in neuronal nuclei imaged from end-stage C9orf72 layer V motor cortex (Fig. 3, Additional file 3: Figure S3). While Lamin B1 invaginations were observed in a subset of postmortem C9orf72 neurons, they were not more common compared to controls (Fig. 3c, Additional file 3: Figure S3C-D) reminiscent of our observations in iPSNs. Consistent with our observations in iPSNs (Figs. 1,2, Additional file 1: Figure S1-2), and 2D analyses in thin paraffin embedded postmortem tissue sections (Fig. 3, Additional file 3: Figure S3), our 3D analyses indicate that nuclear sphericity, volume, and the frequency of Lamin B1 invaginations were similar in nuclei isolated from control and *C9orf72* postmortem motor cortex (Fig. 4, Additional file 4: Figure S4). Collectively, these data suggest that altered nuclear morphology and nuclear lamina invaginations are not a unique pathological feature of C9orf72 ALS/FTD.

## Discussion

The proper interplay between the nuclear lamina and NPCs is essential for coordinating overall nuclear organization, structure, and function [9, 16]. Recent studies provide multiple lines of evidence that NPC and NCT abnormalities can result from the C9orf72 HRE [3, 7, 10, 14, 36]. However, to date, overall nuclear morphology has yet to be thoroughly examined in disease relevant cell types. Despite prior work examining the nuclear lamina in G_4_C_2_ and DPR overexpression based model systems [18, 37], which concluded that there were nuclear membrane alterations in those models, our current analyses using a large number of iPSC lines and postmortem patient tissues suggest that there are in fact no overt alterations to overall nuclear morphology or the frequency of Lamin B1 invaginations in C9orf72 ALS/FTD. While a previous study in postmortem tissue characterized nuclear morphology on the basis of “abnormal” vs “normal” [29], here we comprehensively evaluated nuclear shape in 2D and 3D using measures of circularity and sphericity in iPSNs differentiated from young and aged iPSCs as well as postmortem motor cortex. Employing our more standardized and robust analysis, the overall conclusion that nuclear morphology is unaltered in C9orf72 ALS/FTD (Figs. 1, 2, 3, 4) is consistent with prior studies [29].

In addition to our global morphology analyses, we examined nuclear lamina pathology in iPSNs and postmortem motor cortex. Based on prior work from diseases caused by mutations in nuclear lamins (laminopathies), nuclear lamina pathology is observed as an invagination of the nuclear envelope [15, 35]. In our current study, invaginations were defined as area where Lamin B1 immunoreactivity extended partway into the nucleoplasm before retreating back towards the “nuclear rim.” Invaginations differ from nuclear folds in that a fold can be observed as an area of lamin staining which extends from one side of the nuclear rim to the other side thereby forming a complete “bridge” across the entirety of the nucleus. In fact, it has been previously described that multiple lamin staining patterns including “rim”, “punctate”, “diffuse”, and “folded” can be observed in both control and C9orf72 ALS/FTD postmortem tissues [29]. Consistent with that prior 2D study in a small number of postmortem patient tissues [29], the nuclear lamina of human neurons appears to form a complex topology with many apparent “folds” along the 3D nuclear surface (Figs. 1a, 2a). While the biological function of this folded lamina patterning is not yet understood, the possibility remains that some previous reports of nuclear lamina disruptions in iPSNs [24] simply reflect the normal folding along the 3D surface and edges of neuronal nuclei. Therefore, it is important to carefully consider whether nuclear lamina abnormalities reported are truly pathological invaginations such as those known to occur in laminopathies [15, 35]. To avoid errors in classification, it is essential to evaluate the entire nucleus in 3D space in order to properly distinguish between an invagination and normal nuclear curvature. Prior studies in both non-neuronal cell lines [1, 7, 18, 37] and postmortem tissues [29] appear to misclassify “normal” nuclear curvatures as “folding” pathology. Despite this misclassification, multiple groups have evaluated 2D images and reported an increase in nuclear folding in C9orf72 ALS/FTD model systems [1, 7, 18, 37]. However, all of these studies were conducted using massive overexpression of the C9orf72 HRE or individual DPRs which often accumulate in the perinuclear space [29, 34]. As nuclear membrane morphology such as overall curvature, invaginations, or folding can be impacted by increased mechanical force from actin fibers [12, 15], it is plausible that massive artificial overexpression triggers an increase in mechanical pressure on the nucleus. In contrast, our current analysis thoroughly examines true nuclear lamina invagination pathology by accurately tracing Lamin B1 fibers in 2D and 3D space in endogenous model systems.

Consistent with previous reports that aged iPSCs display nuclear lamina alterations reminiscent of those observed in laminopathies and cellular senescence [25], we found that Lamin B1 invaginations increased with iPSC passage number in both control and C9orf72 iPSNs. Interestingly, NCT capacity and the expression of nuclear transport proteins decline with age [4, 22, 23, 26] and inhibition of nuclear import can impact nuclear lamins [13]. Therefore, a plausible hypothesis is that increased frequency of Lamin B1 invaginations is related to normal age related decline in NCT. Nonetheless, these studies, as well as our own, highlight the critical importance of matching passage number between cell lines when examining nuclear biology in iPSC based neurodegenerative disease models.

## Conclusions

Collectively, our data suggest that Lamin B1 invaginations are commonly observed in human neurons. However, they do not appear to be more frequent in the context of the C9orf72 HRE. Instead, the occurrence of these morphological alterations increases with cellular age independent of the C9orf72 mutation.

## Supplementary Information

**Additional file 1. Figure 1**, Related to Figure 1: Additional graphical representations of data in Figure 1. (A) Alternative graphical representation of quantification of nuclear sphericity. n = 8 control and 8 C9orf72 iPSC lines, at least 100 Map2+ neurons per line and iPSC passage. Each data point represents the average of 100 cells per iPSC line. (B) Graphical representation of nuclear sphericity measurements from each individual iPSC line. (C) Alternative graphical representation of quantification of nuclear volume. n = 8 control and 8 C9orf72 iPSC lines, at least 100 Map2+ neurons per line and iPSC passage. Each data point represents the average of 100 cells per iPSC line. Two-way ANOVA Tukey’s multiple comparison test was used to calculate statistical significance. * p < 0.05, ** p < 0.01. (D) Graphical representation of nuclear volume measurements from each individual iPSC line. (E) Alternative graphical representation of quantification of number of Lamin B1 invaginations per nucleus. n = 8 control and 8 C9orf72 iPSC lines, at least 100 Map2+ neurons per line and iPSC passage. Each data point represents the average of 100 cells per iPSC line. Chi-square test was used to calculate statistical significance. **** p < 0.0001. (F) Graphical representation of number of Lamin B1 invaginations per nucleus from each individual iPSC line. Chi-square test was used to calculate statistical significance. **** p < 0.0001.

**Additional file 2. Figure 2**, Related to Figure 2: Additional graphical representations of data in Figure 2. (A) Alternative graphical representation of quantification of nuclear sphericity. n = 10 control and 10 C9orf72 iPSC lines, at least 50 NeuN+ nuclei per line. Each data point represents the average of 50 nuclei per iPSC line. (B) Graphical representation of nuclear sphericity measurements from each individual iPSC line. (C) Alternative graphical representation of quantification of nuclear volume. n = 10 control and 10 C9orf72 iPSC lines, at least 50 NeuN+ nuclei per line. Each data point represents the average of 50 nuclei per iPSC line. (D) Graphical representation of nuclear volume measurements from each individual iPSC line. (E) Alternative graphical representation of quantification of number of Lamin B1 invaginations per nucleus. n = 10 control and 10 C9orf72 iPSC lines, at least 50 NeuN+ nuclei per line. Each data point represents the average of 50 nuclei per iPSC line. (F) Graphical representation of number of Lamin B1 invaginations per nucleus from each individual iPSC line.

**Additional file 3. Figure 3**, Related to Figure 3: Additional graphical representations of data in Figure 3. (A) Alternative graphical representation of quantification of nuclear circularity. n = 8 control and 8 C9orf72 patients, at least 100 Map2+ neurons per case. Each data point represents the average of 100 neurons per case. (B) Graphical representation of nuclear circularity measurements from each individual patient. (C) Alternative graphical representation of quantification of number of Lamin B1 invaginations per nucleus. n = 8 control and 8 C9orf72 patients, at least 100 Map2+ neurons per case. Each data point represents the average of 100 neurons per case. (D) Graphical representation of number of Lamin B1 invaginations per nucleus from each individual patient.

**Additional file 4. Figure 4**, Related to Figure 4: Additional graphical representations of data in Figure 4. (A) Alternative graphical representation of quantification of nuclear sphericity. n = 4 control and 4 C9orf72 patients, at least 50 NeuN+ nuclei per case. Each data point represents the average of 50 nuclei per case. (B) Graphical representation of nuclear sphericity measurements from each individual patient. (C) Alternative graphical representation of quantification of nuclear volume. n = 4 control and 4 C9orf72 patients, at least 50 NeuN+ nuclei per case. Each data point represents the average of 50 nuclei per case. (D) Graphical representation of nuclear volume measurements from each individual patient. (E) Alternative graphical representation of quantification of number of Lamin B1 invaginations per nucleus. n = 4 control and 4 C9orf72 patients, at least 50 NeuN+ nuclei per case. Each data point represents the average of 50 nuclei per case. (F) Graphical representation of number of Lamin B1 invaginations per nucleus from each individual patient.

**Additional file 5.** Table 1 and 2, Related to Materials and Methods: Demographic information for iPSC lines and postmortem tissues used in this study.

## Data Availability

All iPSC lines are available from the Cedars Sinai Answer ALS cell line bank (https://www.cedars-sinai.edu/Research/Research-Cores/Induced-Pluripotent-Stem-Cell-Core-/Answer-ALS-Project.aspx) or through the Answer ALS Data portal (https://dataportal.answerals.org/home).
