# Supplementary figures and images for: Nuclear lamina invaginations are not a pathological feature of C9orf72 ALS/FTD
Source: Acta Neuropathol Commun. 2021 Mar 19;9:45. doi: 10.1186/s40478-021-01150-5 (PMC7977268; doi:10.1186/s40478-021-01150-5)

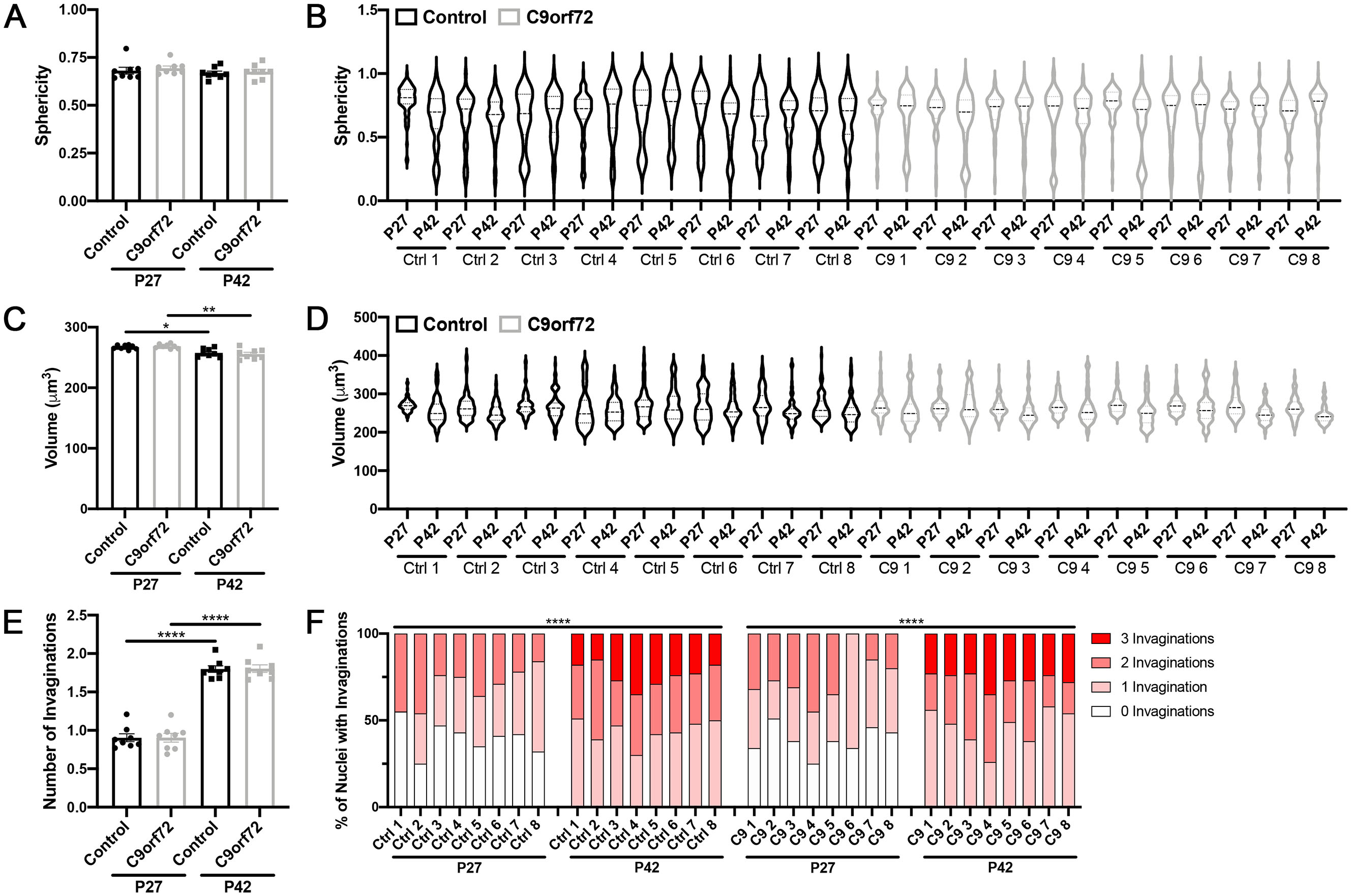

Supplement: Supplementary file 1 — Additional file 1. Figure 1, Related to Figure 1: Additional graphical representations of data in Figure 1. (A) Alternative graphical representation of quantification of nuclear sphericity. n = 8 control and 8 C9orf72 iPSC lines, at least 100 Map2+ neurons per line and iPSC passage. Each data point represents the average of 100 cells per iPSC line. (B) Graphical representation of nuclear sphericity measurements from each individual iPSC line. (C) Alternative graphical representation of quantification of nuclear volume. n = 8 control and 8 C9orf72 iPSC lines, at least 100 Map2+ neurons per line and iPSC passage. Each data point represents the average of 100 cells per iPSC line. Two-way ANOVA Tukey’s multiple comparison test was used to calculate statistical significance. * p < 0.05, ** p < 0.01. (D) Graphical representation of nuclear volume measurements from each individual iPSC line. (E) Alternative graphical representation of quantification of number of Lamin B1 invaginations per nucleus. n = 8 control and 8 C9orf72 iPSC lines, at least 100 Map2+ neurons per line and iPSC passage. Each data point represents the average of 100 cells per iPSC line. Chi-square test was used to calculate statistical significance. **** p < 0.0001. (F) Graphical representation of number of Lamin B1 invaginations per nucleus from each individual iPSC line. Chi-square test was used to calculate statistical significance. **** p < 0.0001. [file 40478_2021_1150_MOESM1_ESM.jpg]

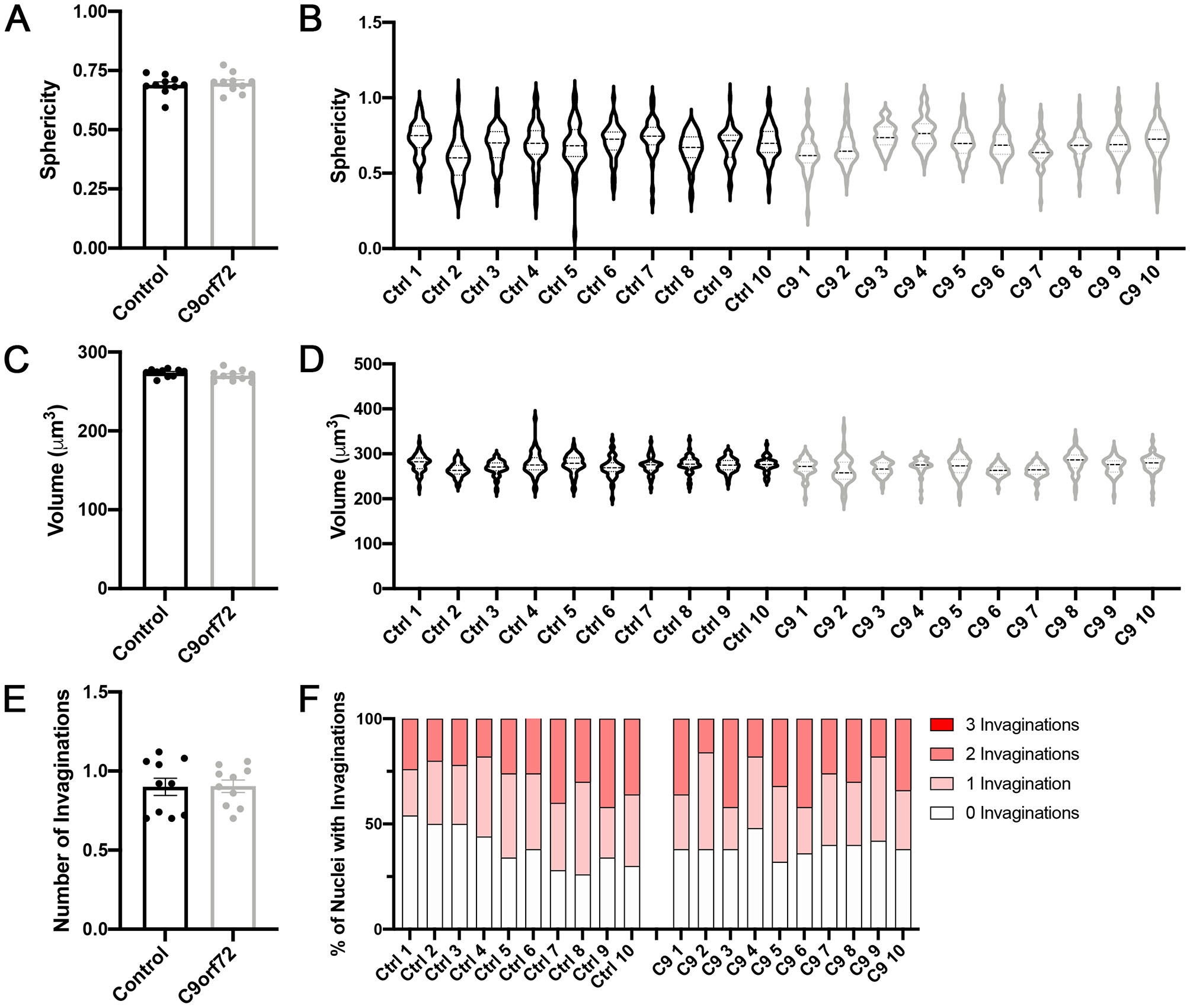

Supplement: Supplementary file 2 — Additional file 2. Figure 2, Related to Figure 2: Additional graphical representations of data in Figure 2. (A) Alternative graphical representation of quantification of nuclear sphericity. n = 10 control and 10 C9orf72 iPSC lines, at least 50 NeuN+ nuclei per line. Each data point represents the average of 50 nuclei per iPSC line. (B) Graphical representation of nuclear sphericity measurements from each individual iPSC line. (C) Alternative graphical representation of quantification of nuclear volume. n = 10 control and 10 C9orf72 iPSC lines, at least 50 NeuN+ nuclei per line. Each data point represents the average of 50 nuclei per iPSC line. (D) Graphical representation of nuclear volume measurements from each individual iPSC line. (E) Alternative graphical representation of quantification of number of Lamin B1 invaginations per nucleus. n = 10 control and 10 C9orf72 iPSC lines, at least 50 NeuN+ nuclei per line. Each data point represents the average of 50 nuclei per iPSC line. (F) Graphical representation of number of Lamin B1 invaginations per nucleus from each individual iPSC line. [file 40478_2021_1150_MOESM2_ESM.jpg]

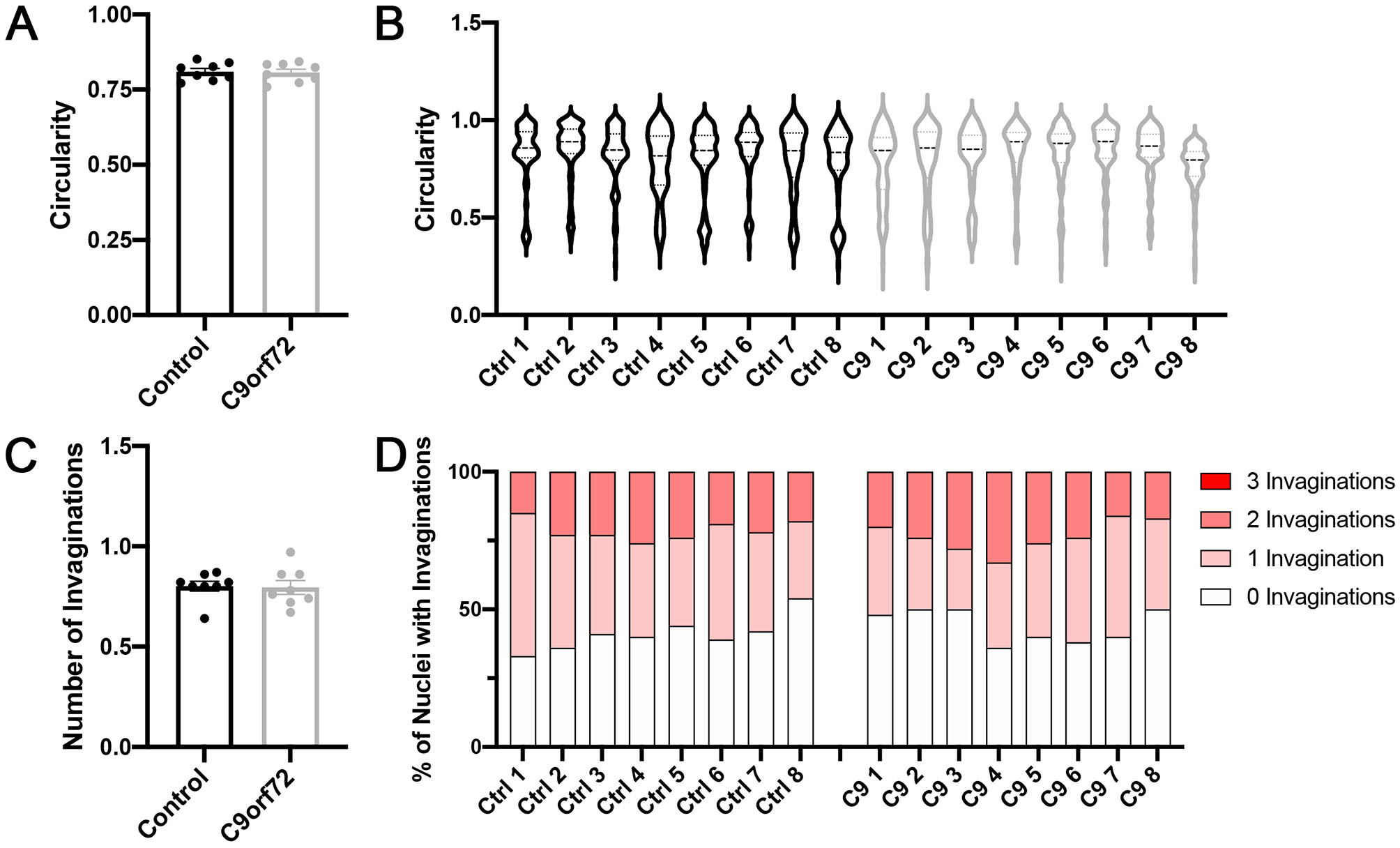

Supplement: Supplementary file 3 — Additional file 3. Figure 3, Related to Figure 3: Additional graphical representations of data in Figure 3. (A) Alternative graphical representation of quantification of nuclear circularity. n = 8 control and 8 C9orf72 patients, at least 100 Map2+ neurons per case. Each data point represents the average of 100 neurons per case. (B) Graphical representation of nuclear circularity measurements from each individual patient. (C) Alternative graphical representation of quantification of number of Lamin B1 invaginations per nucleus. n = 8 control and 8 C9orf72 patients, at least 100 Map2+ neurons per case. Each data point represents the average of 100 neurons per case. (D) Graphical representation of number of Lamin B1 invaginations per nucleus from each individual patient. [file 40478_2021_1150_MOESM3_ESM.jpg]

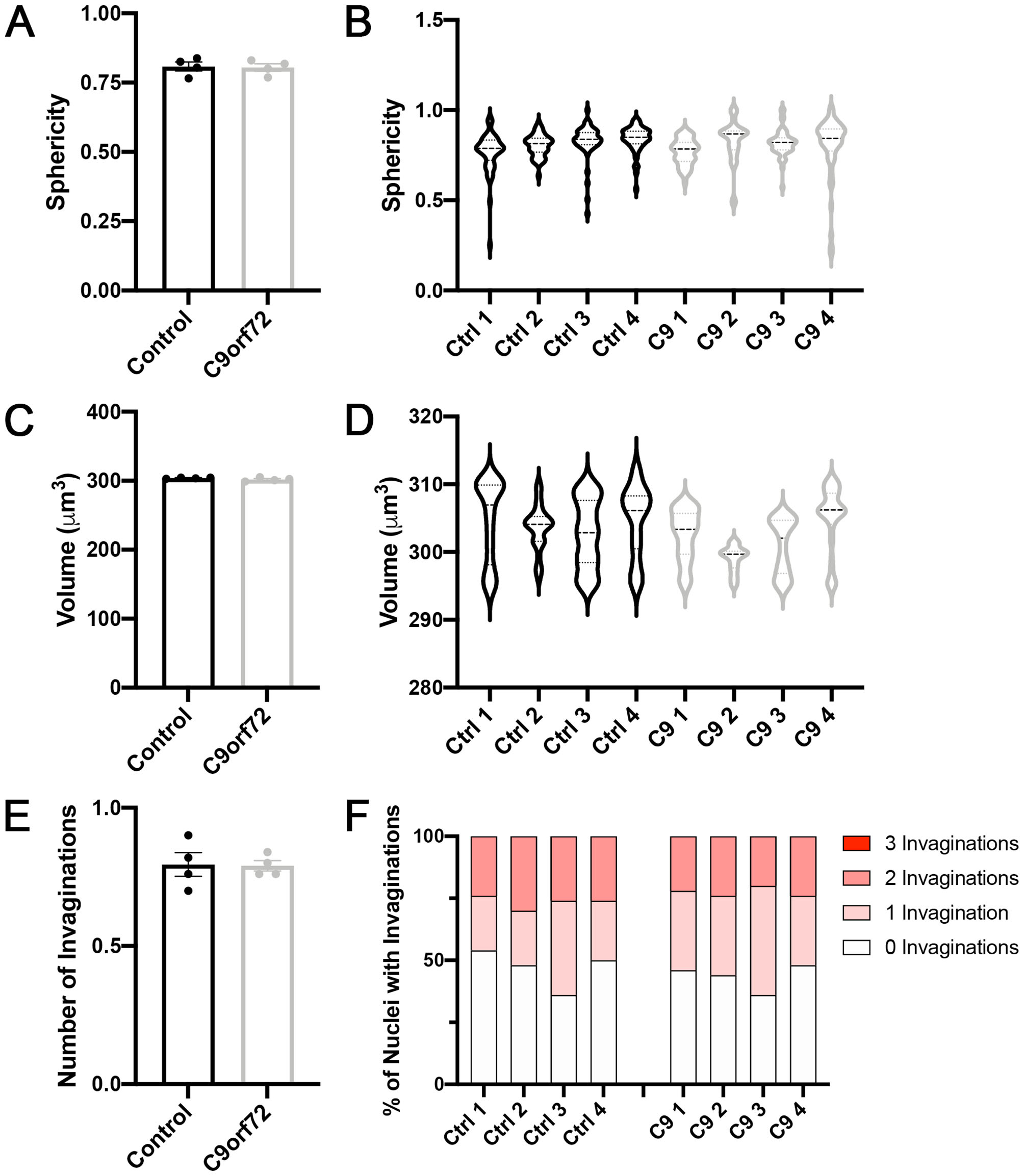

Supplement: Supplementary file 4 — Additional file 4. Figure 4, Related to Figure 4: Additional graphical representations of data in Figure 4. (A) Alternative graphical representation of quantification of nuclear sphericity. n = 4 control and 4 C9orf72 patients, at least 50 NeuN+ nuclei per case. Each data point represents the average of 50 nuclei per case. (B) Graphical representation of nuclear sphericity measurements from each individual patient. (C) Alternative graphical representation of quantification of nuclear volume. n = 4 control and 4 C9orf72 patients, at least 50 NeuN+ nuclei per case. Each data point represents the average of 50 nuclei per case. (D) Graphical representation of nuclear volume measurements from each individual patient. (E) Alternative graphical representation of quantification of number of Lamin B1 invaginations per nucleus. n = 4 control and 4 C9orf72 patients, at least 50 NeuN+ nuclei per case. Each data point represents the average of 50 nuclei per case. (F) Graphical representation of number of Lamin B1 invaginations per nucleus from each individual patient. [file 40478_2021_1150_MOESM4_ESM.jpg]
