# Supplementary material for: Nuclear lamina invaginations are not a pathological feature of C9orf72 ALS/FTD
Source: Acta Neuropathol Commun. 2021 Mar 19;9:45. doi: 10.1186/s40478-021-01150-5 (PMC7977268; doi:10.1186/s40478-021-01150-5)
Supplement: Supplementary file 5 — Additional file 5. Table 1 and 2, Related to Materials and Methods: Demographic information for iPSC lines and postmortem tissues used in this study. [file 40478_2021_1150_MOESM5_ESM.docx]

**Supplemental Table 1: Demographic Information for iPSC Lines**

| **iPSC Line Name** | **Source** | **Clinical Diagnosis** | **Age at Time of Collection** | **Sex** | **Origin** |
| --- | --- | --- | --- | --- | --- |
| EDi036-A | Cedars-Sinai | Non-neurologic control | 79 | Female | PBMC |
| EDi037-A | Cedars-Sinai | Non-neurologic control | 79 | Male | PBMC |
| EDi029-A | Cedars-Sinai | Non-neurologic control | 80 | Male | PBMC |
| EDi034-A | Cedars-Sinai | Non-neurologic control | 79 | Female | PBMC |
| CS1ATZ | Cedars-Sinai | Non-neurologic control | 60 | Male | PBMC |
| CS8PAA | Cedars-Sinai | Non-neurologic control | 58 | Female | PBMC |
| EDi043-A | Cedars-Sinai | Non-neurologic control | 80 | Male | PBMC |
| CS0002 | Cedars-Sinai | Non-neurologic control | 51 | Male | PBMC |
| CS9XH7 | Cedars-Sinai | Non-neurologic control | 53 | Male | PBMC |
| CS0BUU | Cedars-Sinai | C9orf72 | 63 | Female | PBMC |
| CS7VCZ | Cedars-Sinai | C9orf72 | 64 | Male | PBMC |
| CS0LPK | Cedars-Sinai | C9orf72 | 67 | Male | PBMC |
| CS6ZLD | Cedars-Sinai | C9orf72 |  | Female | PBMC |
| CS8KT3 | Cedars-Sinai | C9orf72 | 60 | Male | PBMC |
| CS2YNL | Cedars-Sinai | C9orf72 | 60 | Male | PBMC |
| CS0NKC | Cedars-Sinai | C9orf72 | 52 | Female | PBMC |
| CS6CLW | Cedars-Sinai | C9orf72 |  | Male | PBMC |
| CS6UC9 | Cedars-Sinai | C9orf72 | 54 | Male | PBMC |
| 59-1 | K. Talbot | Isogenic Correction of OXC9-02 | 62 | Female | Fibroblast |
| OXC9-02-02 | K. Talbot | C9orf72 | 62 | Female | Fibroblast |

**Supplemental Table 2: Demographic Information for Postmortem Human Tissue**

|  | **Clinical Diagnosis** | **Age of Death** | **Sex** |
| --- | --- | --- | --- |
| **Control** | Non-neurologic control | 70 | Female |
| **Control** | Non-neurologic control | 92 | Female |
| **Control** | Non-neurologic control | 72 | Male |
| **Control** | Non-neurologic control | 37 | Female |
| **Control** | Non-neurologic control | 50 | Male |
| **Control** | Non-neurologic control | 52 | Male |
| **Control** | Non-neurologic control | 74 | Female |
| **Control** | Non-neurologic control | 71 | Female |
| **C9orf72** | C9orf72 ALS/FTD | 59 | Male |
| **C9orf72** | C9orf72 ALS | 72 | Male |
| **C9orf72** | C9orf72 ALS | 69 | Female |
| **C9orf72** | C9orf72 ALS/FTD | 61 | Female |
| **C9orf72** | C9orf72 ALS | 68 | Female |
| **C9orf72** | C9orf72 ALS | 61 | Female |
| **C9orf72** | C9orf72 ALS | 51 | Female |
| **C9orf72** | C9orf72 ALS/FTD | 74 | Male |
